# Draft Genome Sequence of *Francisella tularensis* Strain 410108 from Tibet, China

**DOI:** 10.1128/genomeA.01489-15

**Published:** 2015-12-17

**Authors:** Lihua Song, Yonghui Yu, Le Feng, Tao Wang, Jun He, Hong Zhu, Qing Duan

**Affiliations:** State Key Laboratory of Pathogen and Biosecurity, Beijing Institute of Microbiology and Epidemiology, Beijing, China

## Abstract

*Francisella tularensis* is the etiological agent of the potentially fatal disease tularemia. Here, we report the draft genome sequence of a virulent human isolate from Tibet, China in 1962, *F. tularensis* strain 410108, an intermediate-genotype strain of *F. tularensis* subsp. *holarctica* between biovar *japonica* and non-*japonica* strains in the world.

## GENOME ANNOUNCEMENT

*Francisella tularensis* is a Gram-negative bacterium and the causative agent of tularemia, a public health issue across the northern hemisphere (1). There are four subspecies of *F. tularensis*: subsp. *tularensis*, *holarctica*, *novicida*, and *mediaasiatica* (2, 3). These four subspecies are closely related genetically and share most biochemical characteristics, but only the first two subspecies are clinically important (4, 5). In China, tularemia or *F. tularensis* has been found in Inner Mongolia, Tibet, Xinjiang, Jilin, Shandong, Heilongjiang, and Qinghai provinces or municipalities (6). Current reports of *F. tularensis* in China belong to subsp. *holarctica*.

Here, we report the draft genome of *F. tularensis* subsp. *holarctica* strain 410108, isolated from a tularemia patient in Dangxiong, Tibet in 1962. This strain exhibits a 50% lethal dose of less than 5 CFU by intraperitoneal infection of either mice or guinea pigs. Genotyping studies demonstrated that this strain belongs to an intermediate genotype between subsp. *holarctica* biovar *japonica* and other subsp. *holarctica* strains in the world. Identification of this intermediate genotype provides evidence for an Asian origin and radiation of *holarctica*-type tularemia.

The strain 410108 was grown on a glucose-cysteine blood agar plate with 5% CO_2_ at 37°C. Cells were harvested by scraping and suspension in PBS followed by treatment with protease K. DNA was extracted using phenol-chloroform-isoamyl alcohol followed by isopropanol precipitation as previously described (7). The DNA was then used for full-length genome sequencing using an Illumina GA genome analyzer (Life Technologies). Two different DNA libraries (400-bp paired end and 3,000-bp mate pair) were sequenced, yielding 26,713,598 and 26,881,845 reads, respectively. *De novo* assembly was performed using SOAPdenovo version 1.05 (8). A total of 302 contigs were generated with an average length of 311,187 bp (*N*_50_ size of 655,460 bp and total size of 1,867,125 bp), and were further assembled into 6 scaffolds. The gaps within the scaffolds were resolved using PCR and Sanger sequencing.

The draft chromosome of strain 410108 is 1,899,676 bp in size. The G+C content is 31.97%. The protein coding sequences (CDSs) were analyzed using FgenesB (SoftBerry), predicting a total of 1,508 CDSs (>90 amino acids). The putative functions of these CDSs were annotated using BLASTp as well as KEGG, COG, and GO protein databases (9, 10). Interestingly, strain 410108 has an intact FTT1080c homolog, which encodes a hypothetical membrane protein and has been recognized as subsp. *tularensis*-specific (11, 12). We surmise that this FTT1080c homolog might be a virulence gene candidate.

This is the first genome report of an *F. tularensis* strain in China. It is also the first genome of an intermediate genotype of *F. tularensis* subsp. *holarctica* between biovar *japonica* and other non-*japonica* strains in the world. Next we will perform genomic comparisons between more genomes of China isolates and genomes of other areas, which will likely provide new insights into the evolution and radiation of tularemia.

### Nucleotide sequence accession number.

The draft genome sequence has been deposited in GenBank under the accession no. CP012092.
